# The influence of “kickstand” screws on the mechanical performance of a lateral distal femoral plate: a finite element analysis

**DOI:** 10.1302/2046-3758.148.BJR-2024-0479.R1

**Published:** 2025-08-19

**Authors:** Alex Trompeter, Alexis Christen, Claus Gerber, Bernhard Hofstaetter, Felix Wipf, Jason Lowe

**Affiliations:** 1 City St George’s University of London, St George’s University Hospital, London, UK; 2 Stryker, Selzach, Switzerland; 3 Stryker Trauma GmbH, Schönkirchen/Kiel, Germany; 4 University of Arizona, Banner - University Medicine, Alvernon Clinic, Tucson, Arizona, USA

**Keywords:** Fracture, Kickstand, Distal femur, Lateral locked plate, distal femoral, Locking screws, distal femoral fractures, mechanical failures, Finite element analysis, Locked plating, bone defect, stiffness, subchondral bone

## Abstract

**Aims:**

Lateral locked plating of distal femoral fractures is widely reported, yet there remains a 9% to 19% incidence of mechanical failure. Obliquely directed “kickstand screws”, from the metaphyseal portion of a plate toward far-sided articular subchondral bone, have been shown to improve construct stiffness. This study explores the impact of kickstand screws in a finite element analysis bone defect model, comparing plate and screw maximum stress and maximum locking screw forces either with or without the addition of kickstand screws.

**Methods:**

A finite element analysis model of a lateral based femoral plate and fracture gap simulation was created, with material and construct data parameters regarding bone material, implant, and composite model identified. The addition of the upper, lower, or both kickstand screws in an anatomical precontoured lateral distal femoral plate were selected as the variables compared against the absence of kickstand screws. Screw and plate principal stresses (MPa) and locking screw mechanism force (N) were measured.

**Results:**

The addition of the upper kickstand screw or both kickstand screws led to an approximate 40% reduction of stress in the metaphyseal hole closest to the fracture. The addition of the lower, upper, and both kickstand screws led to a 23%, 32%, and 34% reduction of maximum stress in the metaphyseal screws, respectively. The addition of the lower kickstand screw led to a 19% reduction, while the upper or both kickstand screws led to a 23% reduction of maximum force experienced by the locking mechanism.

**Conclusion:**

The addition of kickstand screws improves the mechanical performance of the construct, with reduced stresses experienced by the plate and metaphyseal screws. Furthermore, the maximum forces on the locking screw mechanism were shown to be significantly reduced, providing a protective effect to the polyaxial locking mechanism of the metaphyseal screw.

Cite this article: *Bone Joint Res* 2025;14(8):713–720.

## Article focus

- Finite element analysis (FEA) of the impact of kickstand screws to a lateral distal femoral plate.

## Key messages

- Addition of kickstand screws reduces the stresses experienced by the metaphyseal screws.
- Addition of kickstand screws reduces the forces experienced by the locking screw mechanisms in adjacent screws.

## Strengths and limitations

- FEA can be limited by the data parameters used.
- This study identifies unique behavioural properties of a lateral distal femoral construct with the addition of kickstand screws. This has potential clinical benefits.

## Introduction

Lateral locked plating of distal femoral fractures is a well-recognized technique and has been extensively reported in the literature. The original Less Invasive Stabilization System (LISS) was one of the earliest dedicated anatomical region-specific locked plates for the distal lateral femur.^1,2^ Despite the evolution of plate technology, and widely reported good radiological and clinical outcomes, there remains a 9% to 19% incidence of mechanical failure in laterally based distal femoral constructs. Risk factors for mechanical failure include C3 fractures, especially in the presence of a large zone of comminution or loss of medial column integrity.^3-6^

The bending moment and varus loading of distal femoral constructs remains a challenge to overcome. Laterally based plates are subject to the highest bending moments as the weightbearing axis passes medial to the lateral plate, subjecting the entire construct to a disadvantageous loading pattern. Early loss of fixation is observed through failure of the screw plate locking mechanism. This failure mode is consistently observed in pure load-bearing constructs, particularly when polyaxial locking screws are used.^7^ Locking screws have been shown to improve fixation in porotic bone but need careful consideration in their application. Use of dual-implant construct (medial plates or nail constructs, or combinations of plates and nails) have been used to reduce risk of implant failure and fracture collapse.^8^ These constructs have a clear mechanical benefit, however they require familiarity with anatomical exposures and techniques that may be outside all surgeons’ routine fracture care, and the lateral plate remains the default fixation implant for even the most complex distal femoral fractures. As such, there is a need to consider how laterally based plates can evolve to overcome their mechanical disadvantage.

Addition of a screw directed obliquely from the metaphyseal portion of the plate toward the far-sided articular subchondral bone, or “kickstand screw”, was first shown to improve fracture stiffness in distal femur fractures stabilized with non-locking condylar buttress plates.^9^ Kickstand screws serve to dissipate the load experienced by the construct, and protect the potential fatigue area of the plate, by supporting the far side metaphyseal bone. The resulting construct is an A-frame construct which is inherently much stronger than a pure cantilevered pattern with all screws in a similar trajectory. Additionally, distal femur oblique kickstand screws can be directed toward the posterior medial condyle, the strongest bone of the distal femoral articular block, and thereby “anchor” the construct to this area.^10^ Kickstand screws have been shown to be of benefit in modern precontoured periarticular locking constructs, including proximal humeral,^11^ distal radial,^12^ proximal femur,^13,14^ and tibial plateau fractures.^15^

This finite element analysis (FEA) study aims to compare the maximum principal stresses measured within the metaphyseal screws, the plate and the highest forces experienced at the locking mechanism (the connection between plate and screw) of a distal femoral laterally based plate construct spanning a bone defect, either with or without the addition of kickstand screws.

## Methods

In order to explore the impact of adding kickstand screws into a laterally based construct with a bone defect representing a significant zone of comminution, an appropriate experimental FEA model was required. Data parameters with regard to the bone material, implant, and composite model were identified based on precedent in vitro testing or accepted material properties, as detailed below. The FEA was developed according to Food and Drug Administration (FDA) guidance concerning computational modelling and simulation (FDA-2021-D-0980)^16,17^ using dedicated modelling software (ANSYS Workbench 2023 R1; ANSYS, USA)

### Implants

The FEA simulation model evaluates the Stryker Pangea Distal Lateral Femur Plate (Stryker, USA), herein referred to as the “plate”. Validation of this FEA simulation model was based on in vitro testing of the plate. This plate is made from titanium alloy: Ti6Al4V (Young’s modulus = 110.4 GPa, Poisson’s ratio = 0.33). The kickstand screws are variable angle locking screws and made of a cobalt-chromium alloy - Co28Cr6Mo (Young’s modulus = 220.0 GPa, Poisson’s ratio = 0.29). The plate incorporates two kickstand screws that aim into the medial/posteromedial femoral condyle from the lateral plate, commencing just proximal to the traditional metaphyseal cluster of screws.

### Bone model (shape, size, geometry)

The bone model used for this simulation was based on a real human skeletal geometry CT scan database (Stryker Orthopaedic Modelling & Analytics (SOMA), Stryker).^18^ Six morphometric parameters were measured from 959 femora to determine mean values of the population: femoral length, curvature, anteversion, CCD-angle (caput-collum-diaphyseal, or head-neck-shaft angle), lever arm distance, and intercondylar distance using the Stryker Anatomy Analysis Tool (SAAT).^18^ The bone model of this study was selected to have the six morphometric measurements as close to the mean values as possible. Parameters such as femoral length, curvature, and CCD-angle of the selected bone deviate less than 1% from the mean values, whereas intercondylar distance had the highest deviation at 9%. In essence, this provided a standardized bone model based on the mean values of 959 femora.

### Bone material

The synthetic bone model used for in vitro testing to simulate cortical bone was a glass pearl reinforced polyamide material PA 3200 GF (EOS GmbH, Germany; Electro Optical System, Young’s modulus = 5.4 GPa, Poisson’s ratio = 0.3), which had the most reproducible and predictable behaviour closest to normal bone.^19,20^ The PA 3200 GF material with femur bone geometry was validated for compression loads up to 2,000 N and torsion loading of ± 20 Nm, allowing for implant failure to occur prior to failure of the simulated bone material. The FEA simulation model was designed to align with in vitro testing that allowed compression and torsional loading of distal femur plating constructs. A cancellous filling was added to the simulated bone model to provide bone contact material for the upper kickstand screw. This cancellous filling is based on the synthetic bone model used for in vitro testing to simulate low-quality bone (osteopenic to osteoporotic) made of polyurethane foam (H200-AT, Vosschemie, Young’s modulus = 0.104 GPa, Poisson’s ratio = 0.3) in the density range of 12 to 15 pcf.^21^

### Plate-bone construct (defect model)

**Fig. 1:**
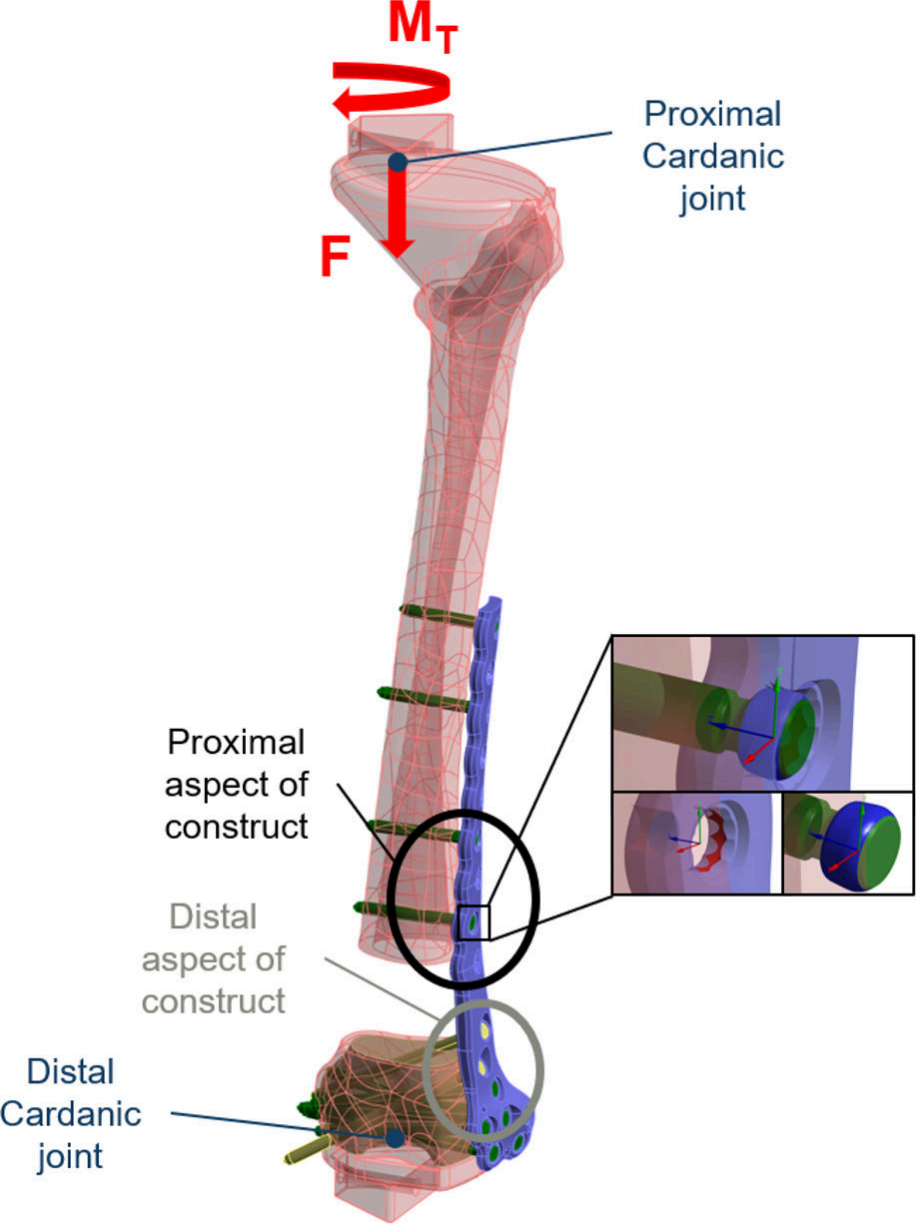
Boundary conditions of the simulated construct based on the biomechanical test setup. A detailed view of the locking screw mechanism between screw head (blue surfaces) and plate (red surfaces) is shown.

**Fig. 2:**
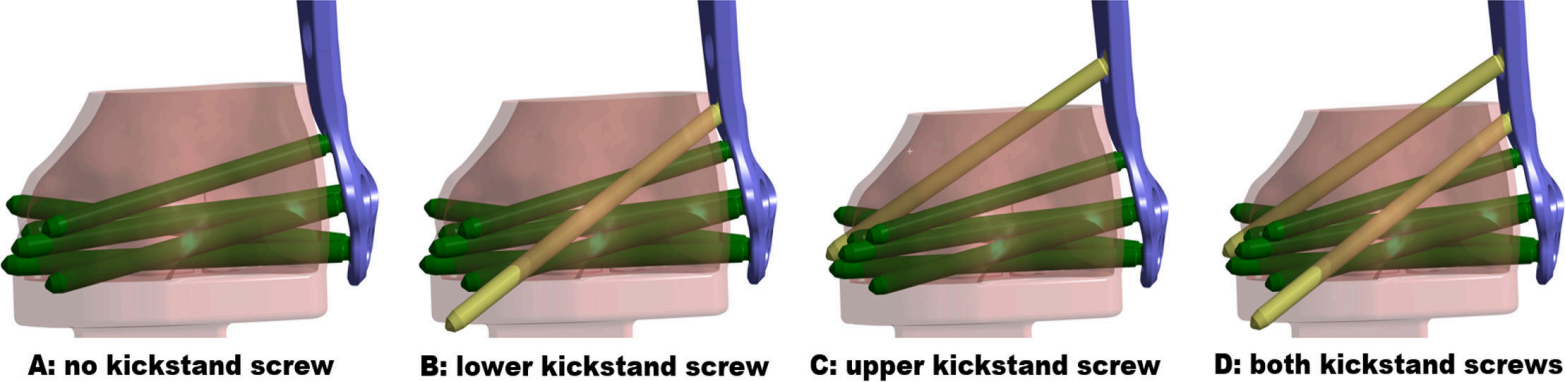
Construct A: without kickstand screws (configuration used for in vitro testing). Construct B: with lower kickstand screw. Construct C: with upper kickstand screw. Construct D: with both kickstand screws.

A 45 mm fracture gap was selected to simulate a large zone of comminution and loss of medial column support (i.e. a AO33-A3 unstable distal fracture). In the FEA simulation model, the plate was offset from the bone by 2 mm as a worst-case condition for plate, and to avoid random contact between plate and bone. For in vitro testing, adapters on the proximal and distal ends of the femur bone provide fixation to Cardanic joints as shown in Figure 1. The boundary conditions of the FEA model simulated the degree of freedom of these Cardanic joints. The variable angle locking mechanism between the plate and a screw was simulated in the FEA model as a joint with the stiffness defined from in vitro testing of the locking mechanism, as shown in Figure 2. FEA simulation models using joints, which allow some flexibility, to represent the variable angle locking mechanism showed more realistic results than modelling total rigid connections.^22^

### Testing metrics

The FEA simulation was modelled to align with in vitro testing that allowed compression and torsional loading of the plate and variable angle locking screws. In accordance with the FDA guidance for 510(k) submissions, the in vitro testing of the plate construct was intended to show substantial equivalence to a predicate device using a mean fatigue limit method according to ASTM STP 731.^16^ ASTM F382^17^ provides guidance for testing metallic bone plates, and rationales for any deviations from this guidance are provided to the FDA. Samples sizes were selected in accordance with ASTM STP 731.^16^ The ratio of compressive and torsional load as well as lever arms were derived from mean maximum loads and worst case loading conditions (including compressive forces, torsion, sagittal bending, and frontal bending moments) based on real anatomical load scenarios derived from published literature.^23-25^ Comparison of the implant failure mode predicted in simulation and confirmed in in vitro testing served as qualitative validation of the FEA simulation model. The mesh convergence study of the FEA simulation model yielded variations below ± 5%. This study compares the maximum principal stress of the implants and the force in the plate-screw interface for different construct configurations.

### Test variables

The construct configurations evaluated in this study are plate construct with either a) no kickstand screws; b) one lower kickstand screw; c) one upper kickstand screw; d) both kickstand screws. Data were collected relating to the maximum principal stresses experienced by the plate and the diaphyseal screw nearest the fracture and all screws in the metaphyseal cluster (measured in MPa), as well as the forces experienced in the locking mechanism (measured in N).

## Results

### Maximum principal plate stresses

**Fig. 3:**
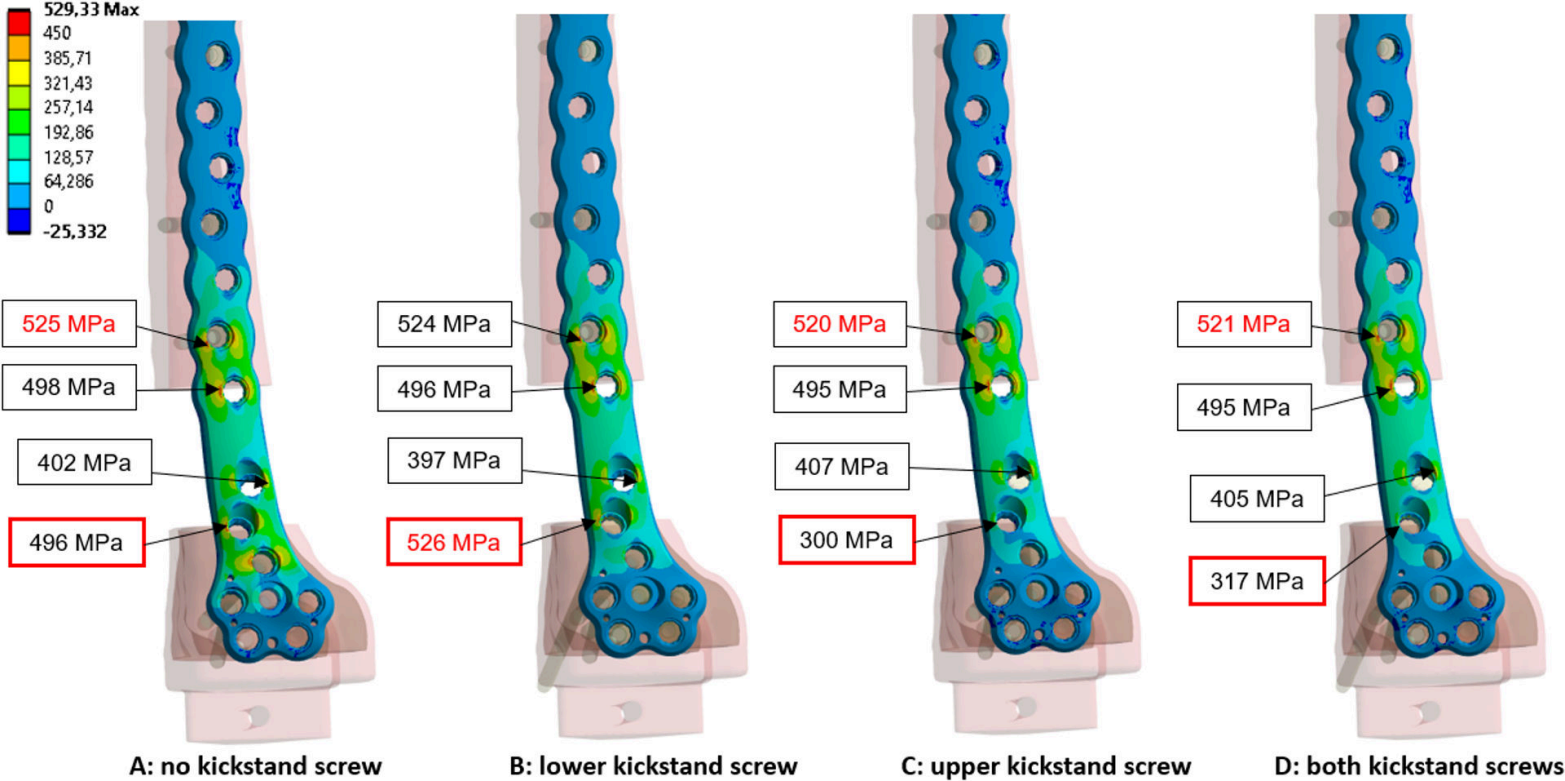
Contour plot of the maximum principal stress (MPa) for the plate implant of all simulated constructs. The text fields show the value and the location of the highest measured stress in the plate. The maximum value is shown in red numbers and the values for the hole where plate breakage occurred during in vitro testing are boxed in red.

Without kickstand screws, the maximum principal stresses in the plate are found in the first and second holes proximal and distal to the fracture, as shown in Figure 3. In vitro testing showed plate breakage in the distal locking hole closest to the fracture highlighted in red in Figure 3; this hole is also referred to as the lower kickstand screw hole. No significant changes in principal stress of the plate shaft proximal to the fracture were observed with the addition of kickstand screws. The addition of the upper kickstand screw or both kickstand screws led to an approximate 40% reduction of stress in the metaphyseal plate hole closest to the fracture (i.e. lower kickstand screw hole) where plate breakage occurred during in vitro testing.

### Maximum principal screw stresses

**Fig. 4:**
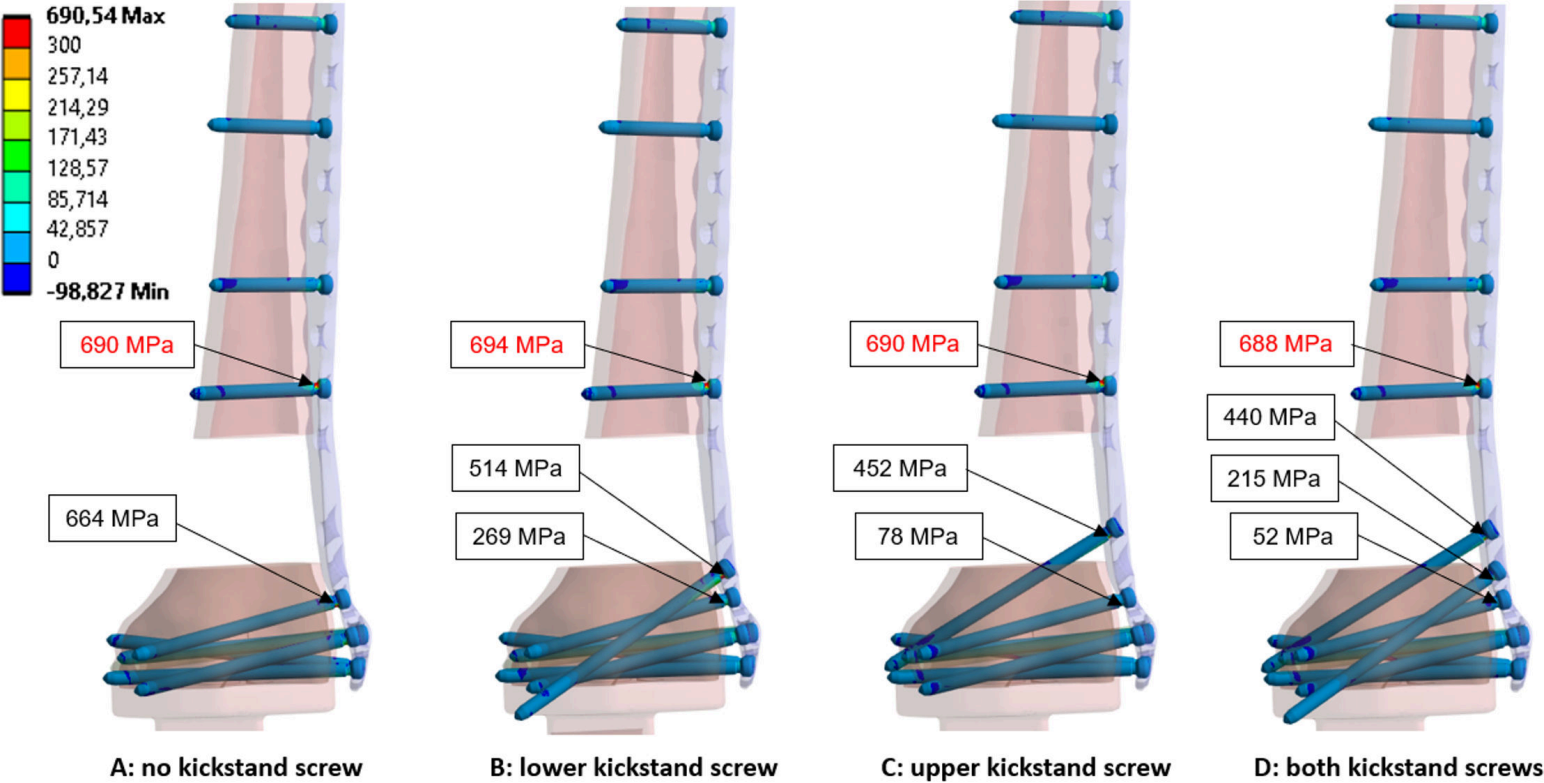
Contour plot of the maximum principal stress (MPa) for the screws of all simulated constructs. The text fields show the value and the location of the highest measured stress in the screws. The maximum value is shown in red numbers.

Without kickstand screws, the maximum principal stresses are found in the neck region of the screws closest to the fracture, both proximal and distal to the fracture, as shown in Figure 4. No change in maximum stress was observed in the shaft screw proximal to the fracture with the addition of kickstand screws. The addition of the lower kickstand screw led to a 23% reduction of maximum stress in the metaphyseal screws. The addition of the upper kickstand screw led to a 32% reduction of maximum stress in the metaphyseal screws. A reduction of 34% was noted with both kickstand screws present.

### Locking screw mechanism forces (screw-plate joint interfaces)

**Fig. 5:**
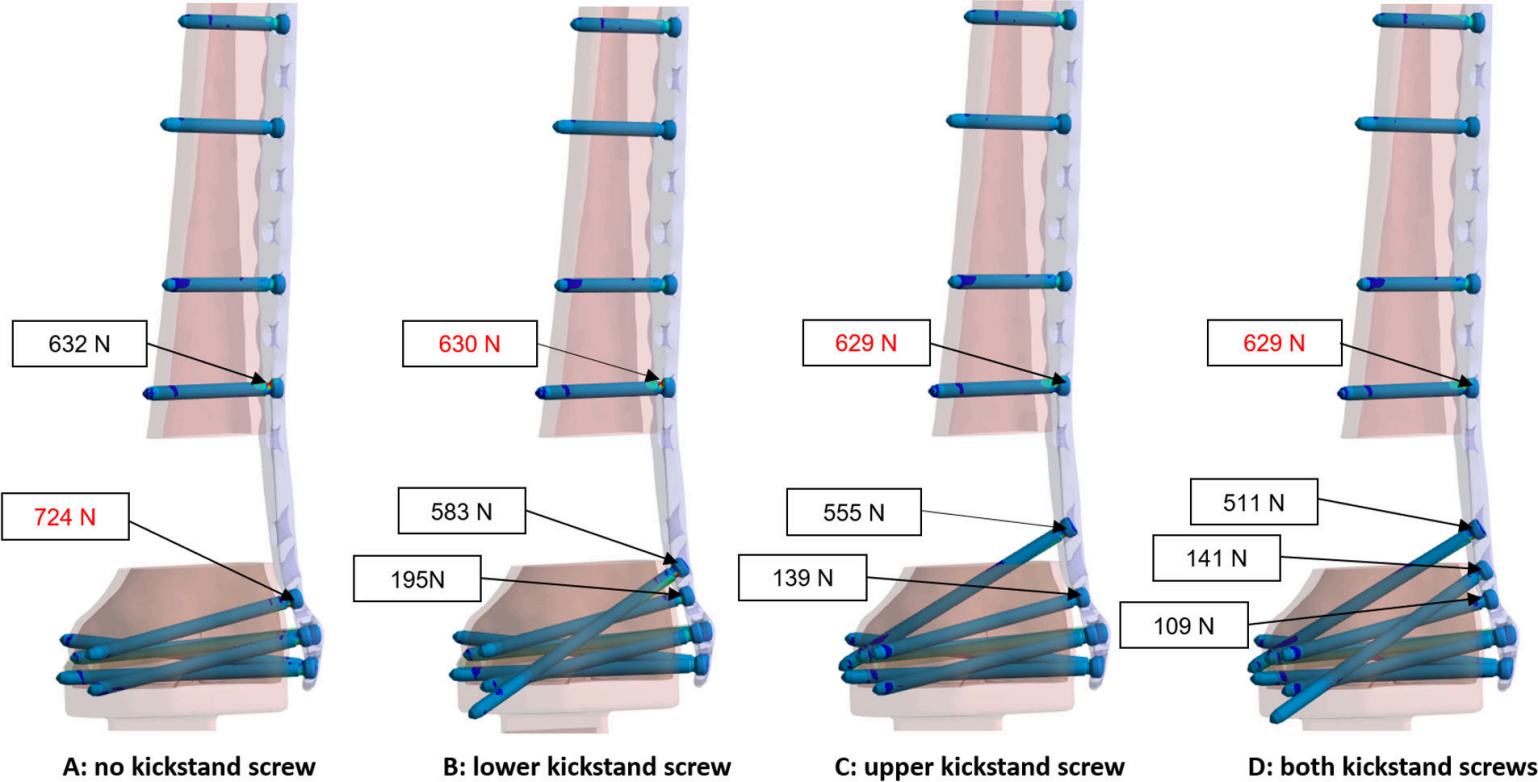
Locking screw mechanism forces measured at the screw-plate joint interfaces for all simulated constructs. The test field shows the value and location of the resultant force (N).

Without kickstand screws, the total forces observed at the locking mechanism were highest for the screws closest to the fracture, both proximal and distal. No change in locking mechanism forces were observed for the shaft screw proximal to the fracture with the addition of kickstand screws. The addition of the lower kickstand screw led to a 19% reduction of maximum forces in the metaphyseal screw locking mechanisms. The addition of the upper kickstand screw led to a 23% reduction of maximum forces in the metaphyseal screw locking mechanisms. Nearly the same reduction was noted with both kickstand screws present (Figure 5).

## Discussion

Technological advances in plate and screw design have increased surgeons’ capacity to successfully treat complex peri- and juxta-articular injuries.^26-28^ Locked plating has become a regular part of the fracture surgeon’s armamentarium, and in distal femoral fractures, the laterally based locked plate remains a common fixation choice. Despite this, there remains a significant incidence of plate failure and nonunion.^29^ Data presented here demonstrate that addition of two kickstand screws decreases stress at the distal plate and distal locking screws, and reduced forces at the distal screw/plate locking interfaces in distal femur fractures with metaphyseal comminution. These data are clinically relevant because early mechanical failure of distal locking screw is a common mode of failure in comminuted, load-bearing, fracture fixation constructs where polyaxial screws are employed.^4-6^

Polyaxial locking screws were developed to enable surgeons to use fixed angled screws that could be directed toward intact bone, and around existing implants, in comminuted short articular segments for native and periprosthetic fractures. The benefits of inserting polyaxial locking screws are tempered by a reduction in cantilever bending load as screw insertion angle increases from 0° to 15°. Lenz et al^30^ observed a statistically significant reduction in load to failure for polyaxial screws inserted at 15°. Similarly, Herbert-Davies et al^31^ reported up to a 43% reduction in locking force to failure between 0° and 15° screw insertion angles. These mechanical studies have potentially significant clinical relevance.^32^

Tank et al^6^ observed that 22% of mechanical failures in poly-axial plate constructs occurred through the locking screw mechanism in distal screws. These findings were supported by Collinge et al^4^ and McDonald et al,^5^ who also observed that failure through the locking screw-plate interface, albeit at a lower incidence of 36% and 20%, respectively. These clinical studies contained a high percentage of C3 (comminuted) fractures (52% to 100%), and each study correlated metadiaphyseal comminution with failure of polyaxial locking screws.

These clinical and biomechanical studies support the need to protect the locking screw mechanism in complex, comminuted distal femur fractures. Distal femur fracture construct augmentation with a second implant has been shown to be biomechanically superior and clinically successful compared to standalone lateral locked constructs.^8^ Dual-implant constructs, however, come at a significant increase in case implant costs, and require knowledge of medial surgical approaches as well as techniques with which not all surgeons may be familiar. Data presented here show that the addition of two kickstand screws decreases stress at the distal plate, distal screw stresses, and the loading of the locking mechanism.

The limitations of this study are those of FEA in general. The FEA model is only as good as the data used to inform it – that is, the properties and parameters of the bone, implant, and construct as a whole are defined using potentially variable information. However, in order to overcome this, data on bone simulation material and implant behaviour for analysis were gleaned from prior work and in vitro testing as part of normal product design and development in line with FDA requirements and regulations.^33,34^ Meeting these standards ensures the validity of the finite element model and that the parameters used sit within acceptable boundaries – these are the same parameters used for regulatory clearance of implants coming to market. There remains no accepted threshold for failure, and as such, parameters required for regulatory clearance have been considered as a suitable threshold here (i.e. benchmarking with an existing device on the market – a so-called ‘predicate device’).

The model used may be seen as artificial – a squared-off bone gap in the metadiaphyseal region is not commonly seen in acute trauma. However, this represents the clinical reality of a large zone of comminution and offers the worst-case scenario for implant loading. A final limitation of FEA and in vitro testing is the absence of the soft-tissue envelope, which can influence the compliance and behaviour of the construct as a whole. It was not possible to add a soft-tissue envelope into this model, as the change in tissue behaviour over time after injury is extremely challenging to replicate and fell outside the scope of this study. Measuring strain at the fracture gap would be perhaps the most clinically relevant parameter, but unfortunately this remains a limitation in FEA modelling as it involves significant variables that cannot be accounted for – patient weight, the nature of the loading and the vector of force application, and the evolution of callus formation during fracture healing. This renders strain as an FEA output as potentially invalid. It is postulated that soft-tissue behaviour would be uniform in any testing model (i.e. the same parameters would be selected) and as such, the implant construct should behave in a similar fashion. The choice therefore of the stresses and forces experienced by the construct can be more clearly defined and more accurately modelled than strain at the fracture site. These represent surrogates for the behaviour of certain predictable aspects of the model in clinical practice.

The results of this study demonstrate that the addition of kickstand screws to a laterally based distal femoral plating construct serve to improve the biomechanical performance of the plate under loading. Firstly, the stresses experienced by the distal plate section itself are reduced if kickstand screws are used. Secondly, the use of kickstand screws reduces the total stresses experienced in the distal metaphyseal screw cluster, providing a protective effect. Thirdly, the kickstands serve to reduce the forces experienced at the locking screw mechanism, which prevents locking mechanism failure in push-out of the screws. In all rounds of testing, the use of the upper kickstand screw exerted a greater effect than the lower kickstand screw, although both either independently or combined served to improve the mechanical environment. The presence of two kickstand screws in this plate design offer surgeons a choice in optimal bone fixation depending on the fracture configuration. The likelihood is that both kickstands would be used in clinical practice.

This study aimed to compare the mechanical properties with regard to maximum stress (as a surrogate for resistance to failure) of a distal femoral laterally based plate construct spanning a bone defect, either with or without the addition of kickstand screws. It has shown that the addition of kickstand screws significantly improved the mechanical performance of the construct, with reduced stresses under loading experienced by the plate and metaphyseal screws. Furthermore, the maximum forces on the locking screw mechanism were shown to be significantly reduced, providing a protective effect to the polyaxial locking mechanism of the metaphyseal screw.

## Data Availability

The data that support the findings for this study are available to other researchers from the corresponding author upon reasonable request.
